# The Relationship Between Combined Lifestyle Behaviors and Mental Illness in Emerging Adults: A Narrative Review

**DOI:** 10.3390/nu18172797

**Published:** 2026-08-27

**Authors:** Noelle Armstrong, Amanda Ciorciari, Kathleen Woolf

**Affiliations:** Department of Nutrition and Food Studies, Steinhardt School of Culture Education and Human Development, New York University, New York, NY 10003, USAkathleen.woolf@nyu.edu (K.W.)

**Keywords:** mental illness, depression, anxiety, lifestyle behaviors, nutrition, physical activity, sleep

## Abstract

Emerging adults (18–25 years) experience the highest prevalence of mental illness, including anxiety and depression, affecting more than 11 million individuals in the United States. Traditional treatment approaches, such as psychotherapy and pharmacotherapy, may not fully address the complex and interrelated risk factors unique to emerging adults. Targeting lifestyle behaviors, including diet, sleep, physical activity, and substance use, has emerged as a promising adjunctive strategy for the prevention and treatment of mental illness. Growing evidence suggests that these behaviors frequently co-occur and may exert synergistic effects on mental health outcomes. This review synthesizes findings from observational studies and randomized controlled trials examining the relationship between combined lifestyle behaviors and mental illness in emerging adults. A total of 17 studies met inclusion criteria, including 14 observational studies and three intervention trials. Observational findings consistently indicated that lifestyle patterns characterized by a greater number of unhealthy behaviors were associated with higher levels of depression, anxiety, and psychological distress. In contrast, the three intervention studies did not demonstrate significant improvements in mental health outcomes following lifestyle interventions. Overall, the findings suggest a synergistic effect of multiple lifestyle behaviors associated with mental illness in emerging adults. However, substantial heterogeneity in how studies defined and categorized “healthy” versus “unhealthy” behaviors limited cross-study comparisons. This review highlights the need for standardized measurement approaches and more rigorous intervention trials to elucidate the potential synergistic effects of combined lifestyle modifications on mental health outcomes in this population.

## 1. Introduction

Mental illness is a serious public health concern, especially among emerging adults (ages 18–25 years), impacting over 11 million individuals in this age group [1]. Common mental illnesses, like anxiety and depression, have increased in recent decades along with related high-risk behaviors, like substance use and abuse, violence, and self-harm [2,3]. Unfortunately, mental illness in emerging adulthood can have widespread negative impacts, including poor academic and job performance, diminished health status, and reduced social connection. If left unaddressed, these issues may last throughout adulthood, leading to serious long-term consequences, including increased morbidity and mortality [3,4]. Chronic conditions, such as cardiovascular disease, diabetes, and obesity, often co-occur with mental illness, which exacerbates the harmful impacts on both physical and mental health [5]. Moreover, the most significant consequence of mental illness is suicide, which remains the second leading cause of death in emerging adults, and in recent years, suicidal ideation, plans, attempts, and deaths have continued to rise [6,7].

The rise in anxiety and depression may be related to the unique pressures experienced among this age group, such as social pressure, academic stresses, and financial challenges [4,8,9]. The increased use of social media also contributes to mental illness, self-harm, and suicidality among emerging adults [10,11]. Despite the expansion of available treatment options, mental illness remains highly prevalent among emerging adults [12]. Furthermore, psychotropic medication and psychotherapy typically address a single area of concern (i.e., physiology or psychology) and may fail to consider a holistic approach. Additionally, psychotropic medications may not be safe or effective long-term [13,14,15,16,17]. Therefore, alternative low-risk and effective strategies to prevent and/or treat mental illness in emerging adults should be examined.

Lifestyle interventions are a promising strategy currently being investigated as an adjunctive treatment for mental illness. An individual’s lifestyle encompasses multiple modifiable risk factors, such as diet quality, sleep duration and quality, physical activity, sedentary behavior, tobacco/nicotine use, and alcohol use, among others. The recent field of “lifestyle psychiatry”, a holistic approach to treatment, focuses specifically on six lifestyle domains, including diet and nutrition, restorative sleep, physical activity, mind–body activities, social connection, and mindfulness and meditation [18]. Lifestyle psychiatry utilizes a multidisciplinary approach to target specific behaviors, like diet, sleep, and physical activity, to improve symptoms of mental illness [19]. Lifestyle interventions have demonstrated greater long-term safety and efficacy compared to medication in reducing symptom severity, particularly for emerging adults [13,14,15,16,20,21].

Lifestyle interventions are especially relevant for emerging adults, a period marked by significant shifts in physical and social environments and new autonomy that directly impact lifestyle behaviors [22,23,24]. Indeed, independent associations between depression and lifestyle behaviors, such as poor diet quality [25,26], inadequate sleep [27,28], physical inactivity [29,30], tobacco/nicotine use [31,32], and alcohol consumption [33], have been established in emerging adults.

Diet quality often declines in emerging adulthood related to increased intake of energy-dense foods, high in saturated fats and sodium [23,34]. Unhealthy dietary behaviors, like meal skipping or irregular mealtimes, are also common among emerging adults [24]. Competing priorities in the form of school, work, and social engagements may lead to skipped meals, irregular mealtimes, or reliance on ultra-processed foods. Poor diet quality has been associated with increased risk of depression in adolescents, emerging adults, and the general adult population [25,26].

Sleep is another behavior that suffers during emerging adulthood [27,28]. Balancing the pressures of academic life and first-time employment as well as nightlife and partying culture in this age group may lead to inadequate sleep. Stress, substance use, and poor mental health, which commonly co-occur among emerging adults, can also contribute to poor sleep quality. Both inadequate sleep and poor sleep quality have been associated with mental illness in young adults [27,28,35].

In addition, more than 50% of emerging adults are physically inactive and are sedentary for an average of 10 h a day, which can negatively impact psychological and physical health [29,30,36]. Emerging adults often engage in seated activities, such as using social media, watching television, playing video games, doing schoolwork and studying, and working an office job, which increases sedentary behavior. Both sedentary behavior and physical inactivity have been linked to mental illness in college-aged adults [29] and across the lifespan [30,36].

Furthermore, substance use and abuse are very prevalent among this age group, which increases the risk for poor health outcomes [37,38]. Alcohol consumption peaks during emerging adulthood, with research estimating that 40–50% surpass the binge threshold each month [37,38]. In addition, the use of and subsequent dependence on tobacco and nicotine products often emerge during this time period [37]. Substance use has been associated with mental illness in emerging adults, with use of substances not only leading to mood disturbances but also being used as a coping mechanism for mental illness [31,32,33].

Overall, the literature suggests that a healthy lifestyle, incorporating a high-quality diet, good sleep quality, less sedentary behavior and more physical activity, and cessation of tobacco/nicotine and alcohol use, may reduce risk of mental illness for the adult population [25,29,31,33,36,39,40]. However, this evidence comes from studies that focus on individual lifestyle behaviors (e.g., diet, sleep, or physical activity alone) rather than combined lifestyle behaviors. While exploring the independent associations between lifestyle behaviors and mental illness provides important information, the recent literature has emphasized the need to explore these behaviors comprehensively to better reflect how these behaviors co-occur in real life [41,42,43,44,45]. Understanding how lifestyle behaviors work together in this population will allow researchers to develop more targeted and effective interventions and inform public health endeavors. To date, no reviews have synthesized the impact of combined lifestyle behaviors on mental illness, specifically within the emerging adult population [46]. Therefore, this review aimed to evaluate and synthesize the relationship between combined lifestyle behaviors and mental illness in emerging adults.

## 2. Methods

This narrative review aimed to summarize the current literature, identify any knowledge gaps, and highlight areas for future research regarding the relationship between combined lifestyle behaviors and mental illness in emerging adults. A literature search was conducted between March 2024 and October 2024 using PubMed, Google Scholar, and Web of Science to identify intervention and observational studies that examined the relationship between combined lifestyle behaviors and mental illness in emerging adults. While PubMed was used as the primary database, Google Scholar and Web of Science were subsequently queried to identify additional studies not captured from the initial PubMed retrieval. The following terms were used in each database: “young or emerging adults,” “lifestyle,” “combined or comprehensive lifestyle,” “lifestyle pattern or cluster,” “mental illness or depression or anxiety,” and “intervention”.

A review protocol ensured all studies assessed combined lifestyle behaviors, as this was the main area of focus. The following inclusion criteria determined the eligibility of observational and/or intervention studies: (1) the primary population was 18–25 year olds, or the results were stratified by age group; (2) the exposure or intervention included diet plus at least one other lifestyle behavior, including sleep, physical activity, alcohol consumption, or tobacco/nicotine use; (3) the study assessed symptoms of common mental illnesses (anxiety or depression) or mental health status (psychological distress, psychiatric diagnosis, psychological well-being); (4) the analysis must have combined lifestyle behaviors to test for a comprehensive effect on the outcome. Studies were excluded if they (1) were non-English articles without a translation available, (2) were a review or qualitative paper, (3) did not include a whole diet intervention but instead focused on a single food or nutrient (i.e., vitamin, supplement, single food intervention), and (4) did not report results (i.e., protocol study). The search was focused on the literature published in the last 10 years to coincide with the increased prevalence of mental illness in emerging adults beginning in the mid 2010s [2]. The literature search results were imported into Covidence for organizational purposes and to assist in study screening [47]. Two reviewers (NA and AC) completed the review process on Covidence. For transparency, the study selection flow diagram is available in the Supplementary Materials (Figure S1).

## 3. Results

### 3.1. Study Characteristics

A total of 17 studies (14 observational studies [48,49,50,51,52,53,54,55,56,57,58,59,60,61] and three interventions [62,63,64]) were included in this review, and their contents are summarized in Table 1. Our review found considerable heterogeneity in the measurement of lifestyle behaviors and mental illness outcomes, which is described below.

### 3.2. Measurement and Analysis of Lifestyle Behaviors

Of the studies included, various tools were used to assess dietary intake. For example, one study conducted physician- or nurse-led screening [56], one study developed a validated questionnaire [51], two studies used 24 h recalls [58,61], two studies used food frequency questionnaires (FFQ) including the Dietary Questionnaire for Epidemiological Studies [50] and the Diet History Questionnaire II (DHQII) [48], while the majority of studies used single questions about specific food groups as a binary representation of healthy foods vs. unhealthy foods, such as fruit and vegetable intake (FVI) [49,52,53,54,55,57,59,60].

A total of 12 observational studies assessed physical activity: one study used the Godin Leisure-Time Exercise Questionnaire [53], two studies created their own physical activity assessment [51,52], four studies measured physical activity using questions from the International Physical Activity Questionnaire (IPAQ) [49,50,58,61], while the remaining five used 1–3 questions to assess physical activity [54,55,57,59,60]. The questions assessed the time per week spent physically active [54,55,57,59]. In addition to measuring physical activity, six studies also assessed sedentary behaviors either using the IPAQ or through a single questionnaire item assessing time spent sitting [49,50,53,58,60,61]. Questions regarding sedentary behavior assessed the time per day spent sitting (e.g., playing video games, using social media, or watching TV) [49,50,53,58,60,61].

In addition to diet and physical activity, sleep was another common behavior assessed in seven observational studies. One of the studies used the Pittsburgh Sleep Quality Index [59] to assess subjective sleep quality and sleep duration. The remaining six studies used original questions about total sleep quantity and sleep quality [48,49,57,58,60,61].

Smoking status, cigarette use, and/or tobacco and nicotine use were measured in 11 studies [48,49,50,51,53,55,56,58,59,60,61]. Alcohol consumption was assessed by 12 studies and included consumption over a period of time [48,50,51,53,54,56,58,59,60,61] and/or binge drinking [49,55,56]. A majority of the studies measured alcohol consumption through self-reported survey questions inquiring about the number of alcoholic beverages consumed within a specific time period [48,49,50,51,53,54,55,58,59,60,61]. One study reported alcohol consumption via a screening interview by a healthcare provider [56].

Additional modifiable behaviors assessed by single studies included marijuana use [50,51,55], recreational drug use [56], and internet addiction [59]. One study also included the impact of adverse experiences on mental well-being as an emerging adult (e.g., verbal violence and emotional neglect) [60]. The assessment and categorization of lifestyle behaviors varied significantly between studies. Table S1 in the Supplementary Materials presents the lifestyle variable cut points used by each study for diet, sleep, physical activity, substance use, and others. As two studies did not use cut points for lifestyle behaviors as their analysis required continuous variables, they are not included in the table [54,57].

Throughout this review, the studies defined a healthy lifestyle as one that is consistent with recommendations for emerging adults for behaviors such as diet, physical activity, sedentary behavior, sleep quality, sleep quantity, and substance use. The opposite would be true for an unhealthy lifestyle: these behaviors are not consistent with the recommendations for emerging adults.

### 3.3. Measurement of Mental Illness

Measurement of mental illness varied widely among both intervention and observational studies. Although the majority of studies assessed mental illness through questionnaires (82%), 19 different questionnaires were used. These tools assessed mental illnesses, like depression and anxiety, as well as psychological distress, stress, mental well-being, and quality of life.

### 3.4. Observational Studies

An individual’s lifestyle encompasses multiple behaviors, which often co-occur. Studying these behaviors together, as a pattern of lifestyle behavior, provides valuable insight into the types of lifestyle patterns that may reduce or increase risk of mental illness. Multiple statistical techniques were used to analyze the association between combined lifestyle behaviors and mental illness. Eight studies conducted latent class analysis (LCA) or latent profile analysis (LPA), identifying classes or profiles that share similar lifestyle behaviors [49,50,53,54,55,58,60,61]. Similarly, two studies used cluster analyses [51,59], two studies used multiple logistic regression [52,56], one study used analysis of variance [48], and one study used hierarchical regression [57].

Some general trends were observed among the 14 observational studies included. Four studies found that a greater number of unhealthy lifestyle behaviors was associated with increased odds of mental illness, while a greater number of healthy lifestyle behaviors was associated with reduced odds of mental illness [48,51,58,59]. For example, Deasy et al. reported that students with high-risk behaviors had higher psychological distress scores, compared to the students with positive health behaviors [51]. Lifestyle behaviors were assessed using a lifestyle behavior questionnaire specifically designed for this study, including self-rated diet quality and physical activity, which may introduce self-report bias [51]. Findings by Xie et al. suggest that healthier lifestyle patterns were associated with lower odds of depression: compared to the “unhealthy lifestyle” group, individuals in all other groups had reduced odds of depression symptoms [58]. In this study, diet quality and depression were examined utilizing the National Health and Nutrition Examination Survey (NHANES), a large national US dataset. They used 24 h dietary recalls to calculate the Healthy Eating Index (HEI) score and assessed depression severity via the validated Patient Health Questionnaire-9 (PHQ-9) [58]. Both Ye et al. and Armstrong et al. found that a greater number of unhealthy lifestyle behaviors was associated with increased odds of anxiety and depression [48,59]. Interestingly, Ye et al. reported that the group of emerging adults with the greater number of unhealthy lifestyle behaviors had a higher proportion of males [59]. Rather than use LCA/LPA, Armstrong et al. grouped individuals by the number of unhealthy lifestyle behaviors, and Ye et al. used clustering, which limits statistical rigor [48,59]. However, both studies incorporated validated assessments of anxiety and depression symptoms. In the assessment of diet, Ye et al. focused solely on fruit and vegetable intake, while, in contrast, Armstrong et al. used food frequency questionnaire data to calculate the HEI, allowing for a more comprehensive assessment of diet [48]. These findings generally support an association between combined lifestyle behaviors and mental illness, although the strength of this evidence varies across studies.

Another trend observed among observational studies was the influence of substance use among lifestyle patterns. Seven LCA/LPA studies reported that lifestyle patterns with high rates of substance use were associated with mental illness [49,50,53,54,55,60,61]. Champion et al. found that “smokers and binge drinkers” had higher levels of psychological distress, depression, and anxiety, while the “inactive, non-smokers” had higher levels of depression when compared to the “moderate risk” class [49]. Similarly, Collins et al. reported that participants in the “high substance use, low physical activity pattern” had increased odds of severe symptoms of depression, anxiety, and stress when compared to the “healthier pattern” [50]. Armstrong et al. found that both males and females in the “pro-inflammatory diet (PID) smokers drinkers” group had increased odds of depression when compared to the “healthy” reference group [61]. Additionally, males in the “poor sleep sedentary drinkers” group and females in the “trouble sleeping” and “PID sedentary” groups had increased odds of depression, suggesting that lifestyle patterns associated with depression may differ by sex [61]. Findings from Dong et al. also highlight substance use as a risky behavior among an unhealthy lifestyle: the “sedentary, substance use, and unhealthy diet” group had higher rates of depression, compared to the “sedentary only” group [53]. However, Dong et al. found the opposite to be true for anxiety, with the “sedentary, substance use, and unhealthy diet” group having lower rates of anxiety, compared to the “sedentary only” group [53]. These findings may be related to how substances are often used by emerging adults as a coping mechanism to temporarily relieve symptoms of anxiety.

In addition to depression, anxiety, and stress, high rates of substance use were also found to be associated with suicidal behaviors and days with poor mental health. Xiang et al. not only included substance use in their analysis but also the social environment of emerging adults such as adverse experiences in the form of verbal violence and emotional neglect [60]. When compared to the “low-risk class”, individuals in the “high-risk class (adverse experience)” had the highest odds of having depression symptoms and suicidal ideation, followed by the “high-risk class (substance abuse/unprotected sex)” [60]. Jao et al. reported that the “high alcohol and drug use” and “low physical activity” groups had higher psychological symptom scores, increased likelihood of reporting a mental health diagnosis, and suicidal and self-injurious behaviors [55]. Grant et al. found that compared to “moderates” and “healthy eaters/exercisers”, the “medium drinkers” and “heavy drinkers” reported more days with poor mental health [54].

These studies were methodologically strong in including a variety of lifestyle behaviors in their LCA/LPA. Although five studies used validated assessment tools to measure similar constructs of mental illness (symptoms of anxiety, depression, and/or psychological stress/distress) [49,50,53,60,61], two did not [54,55], thus limiting comparability across studies. Furthermore, four studies measured only fruit and vegetable intake [49,53,54,55], and one only assessed breakfast, fast food, sweetened beverages, milk, soymilk and yogurt intake [60], leading to a limited understanding of overall diet. However, two studies assessed overall diet quality using the DASH diet score [50] and HEI [61]. These considerable differences in methodology for mental illness and diet make it difficult to compare results and draw generalizable conclusions.

The remaining three observational studies examined specific behaviors that were associated with mental illness among multiple lifestyle behaviors [52,56,57]. Although these studies presented some inconsistent findings, one lifestyle behavior, physical activity, was common among these studies. In particular, two studies reported that PA was associated with psychological distress and depression [52,57]. Using both food consumption and PA variables, Debbia et al. reported that only the frequency of fresh fruit and breads and cereals, the number of hours spent sitting, and intense PA were associated with psychological distress among 12 different food consumption variables and eight PA variables [52]. In another study, Tran et al. examined diet, smoking, alcohol consumption, and recreational drug use, and only diet was found to be associated with depression [56]. Specifically, eating junk food and “bad dietary behavior”, which was defined as irregular rhythm or unbalanced meals, were associated with increased odds of depression [56]. In contrast, Wickham et al. did not find that diet (i.e., raw FVI, processed FVI, fast food, sweets, soda intake) predicted depression but instead found that sleep quality, sleep quantity, and PA were the strongest predictors of depression among lifestyle behaviors [57]. However, this study also measured flourishing, a measure of mental well-being, and found raw FVI to be a predictor of this measurement of well-being [57]. While Debbia et al. included a variety of dietary variables, only diet and physical activity were included in their assessment of lifestyle [52]. In contrast, Tran et al. and Wickham et al. included only a few dietary variables but also measured substance use and physical activity, leading to a more comprehensive assessment of lifestyle [56,57]. All three studies had strong assessments of mental illness, with Debbia et al. and Wickham et al. using validated self-reported symptom screeners and Tran et al. employing in-person, physician-led symptom screening [52,56,57]. Finally, these three studies incorporated multiple logistic regression and hierarchical regression, enabling an exploration of the interactive effects of lifestyle behaviors on symptoms of mental illness. However, these statistical approaches limit our ability to explore how lifestyle behaviors co-occur as one pattern to influence symptoms of mental illness.

### 3.5. Randomized Controlled Trials

Few RCTs have examined the impact of healthy lifestyle changes on mental illness. The three RCTs of lifestyle interventions reported no changes in symptoms of mental illness by the end of the study [63,64,65]. However, one study found a significant increase in quality of life for those receiving the lifestyle intervention compared to the control group [64]. Characteristics of lifestyle interventions varied between trials, including overall intervention topics, session duration, session frequency, and mode of delivery, making it difficult to compare results and draw generalizable conclusions.

The first RCT evaluated the short-term effects of a predominantly physical activity-focused lifestyle intervention on mental health outcomes among emerging adult men. This trial included a 3-month intervention pilot (the HEYMAN program) that utilized weekly 1 h face-to-face sessions that focused on increasing physical activity with some education on healthy eating and stress relief [63]. Researchers assessed mean change in salivary cortisol, depression, anxiety, stress, psychological distress, mental health, and quality of life as secondary outcomes from baseline to 3 months in both the intervention group and the control group [63]. The control group received no education, and participants were asked to continue their usual lifestyle behaviors [63]. The authors found no significant differences in mental health outcomes between the intervention group and the control group [63].

The second RCT also examined the short-term effects of a lifestyle intervention on depression severity and quality of life. Duan et al. conducted an 8-week web-based lifestyle intervention that included weekly 20 min sessions focusing on physical activity and healthy eating (fruit and vegetable intake) [64]. No significant differences were found in depression severity between the intervention and the control groups [64]. However, this study did report a significant increase in quality of life in the intervention group compared to the control group.

The final trial explored the long-term effects of a lifestyle intervention on depression and psychological well-being [65]. This 20-year early prevention trial started in childhood and included a twice yearly dietary intervention and, as children got older, counseling on smoking prevention, increasing physical activity, and avoiding drugs and alcohol, compared to a standard healthcare education control [65]. Researchers assessed depression and psychological well-being as secondary outcomes at a 20-year psychological follow-up and found no differences between the intervention and the control groups [65].

While RCTs provide stronger evidence for causal relationships than observational studies, the methodological limitations of the present studies should be considered when interpreting their findings. Interventions by Ashton et al. and Duan et al. had short durations as well as smaller sample sizes [62,63], while the long-term trial by Kaseva et al. experienced substantial loss to follow-up [64], which can limit the ability to draw firm conclusions about sustained effects. Furthermore, use of self-reported measures, low intervention engagement in some studies, and selective samples, including predominantly male or highly educated participants, may limit the generalizability of the findings. Finally, the lack of significant findings of these trials may also be related to low severity of baseline depression or insufficient intensity of the intervention.

## 4. Discussion

### 4.1. Observational Studies

Of the observational studies included, all 14 of the studies reported significant associations between lifestyle patterns and measures of mental illness. Overall, lifestyle patterns with a greater number of unhealthy behaviors were more likely to be associated with symptoms of mental illness. Because lifestyle behaviors often co-occur with one another, the emergence of lifestyle patterns with multiple unhealthy behaviors in this review was anticipated. Each individual lifestyle behavior included in this review (diet, physical activity and sedentary behavior, sleep quality and quantity, and substance use) has been independently associated with mental illness. Our findings suggest a synergistic effect in which the combination of unhealthy lifestyle behaviors may be associated with an increased burden of mental illness. This finding aligns with a recent meta-analysis that found an association between combined lifestyle behaviors and depression in the general adult population [46]. In this study, individuals with healthier lifestyles had reduced risk of depression [46]. Understanding how lifestyle behaviors co-occur and are associated with mental illness can help guide the development of future interventions.

Multiple observational studies in this review reported that symptoms of mental illness were associated with unhealthy lifestyle patterns that had low rates of physical activity and/or high rates of substance use [50,51,54,55,56,62]. The relationship between physical activity and mental illness, specifically depression and anxiety, has been well documented, and physical activity has been increasingly recommended by practitioners as part of the treatment plan for these conditions [29,30,66]. The research literature implicates multiple mechanisms underlying this relationship, including increased availability of neurotransmitters like serotonin and dopamine, reduced inflammation, and increased motivation [66,67]. The relationship between substance use and mental illness is also well established, as mental illnesses like anxiety and depression often occur comorbidly with substance use disorder [33,37,68]. Many substances, both stimulants and depressants, can cause periods of anxious or depressed moods and may also be used as coping mechanisms for mental illnesses [68].

As previously discussed, emerging adults experience unique pressures that make it particularly difficult to engage in a healthy lifestyle [4,8,9]. Although public health messaging emphasizes the benefits of physical activity and the harms of substance use, emerging adults find it challenging to adopt these healthy lifestyle behaviors [40,69]. Many emerging adults do not have the time or resources to safely engage in adequate physical activity. Physical activity was previously incorporated into their academic curricula in the form of physical education in high schools. Now, emerging adults are expected to continue this behavior with no guidance on how to feasibly incorporate daily physical activity into their new environments that are increasingly sedentary (doing schoolwork and studying, working an office job, spending leisure time using social media, watching television, or playing video games). Furthermore, the social environment of emerging adults often centers around using substances. Social connectedness is increasingly important in this age group, but emerging adults may find it challenging to connect with peers without substances. These substances are also increasingly available and marketed to young people. Overall, the particular challenges faced by emerging adults require unique solutions that provide additional support for the transition from adolescence to adulthood.

Because many lifestyle behaviors have a bidirectional relationship with mental illness, these cross-sectional findings should consider the possibility that mental illness and poor mental health may also lead to increased engagement in unhealthy lifestyle behaviors. Additionally, the potential for residual confounding should be considered given that the covariates included varied widely. Seven of the 14 observational studies adjusted for factors such as age, race, education level, BMI, and socioeconomic status [50,56,57,58,59,60,61]. Thus, other confounding variables, including chronic health conditions, traumatic experiences, family circumstances, and social support, were not consistently controlled for in the study analyses. As a result, these factors may have contributed to some of the observed associations, making it difficult to determine the extent to which lifestyle behaviors themselves were associated with mental health outcomes.

### 4.2. Randomized Controlled Trials

The three RCTs included in this review found no significant differences in depression, anxiety, or psychological distress after a lifestyle intervention. Notably, none of these studies assessed mental illness as a primary outcome. Therefore, to further explore these outcomes, more research on lifestyle interventions to address mental illness should be conducted. However, emerging adults are a very difficult population to recruit and retain [63,64,65,70,71,72]. The trials included in this review cited high dropout rates and loss to follow-up as main study limitations, which may explain the lack of findings [63,64,65]. Duan et al. reported dropout rates of 31.6% from baseline to the end of the intervention and 57.9% from the intervention to 1-month follow-up, while Kaseva et al. reported that approximately half of the original cohort was lost to follow-up [64,65]. Few studies have explored the feasibility and acceptability of these lifestyle interventions in emerging adults with mental illness [73,74,75]. Among the research that does exist, a few themes have been identified [73,74,75]. These themes include (1) stressors and competing responsibilities are attendance and engagement barriers [73,74,75], (2) mental illness may cause functional limitations that make participation in a lifestyle intervention difficult [74,75], and (3) the need for individual support to develop coping mechanisms as well as social support for a successful intervention [73,75]. To better develop acceptable and feasible interventions, future studies should address existing barriers and explore the perspectives of emerging adults to expand on this literature.

One trial reported a significant improvement in quality of life despite observing no change in symptoms of mental illness [64]. Quality of life and mental illness address two different domains and yet are closely connected. Individuals with mental illness tend to have a lower quality of life, related to the burden of mental illness on daily functioning. Compared to the other two interventions, the lifestyle intervention developed by Duan et al. was unique because it addressed social support and coping mechanisms in addition to education on PA and FVI [64]. These findings suggest that a short-term, holistic intervention addressing lifestyle behaviors, including social support and coping mechanisms, could improve an individual’s quality of life.

### 4.3. Strengths and Limitations

Grouping lifestyle behaviors allows us to understand how an overall lifestyle is related to mental illness. This approach is critical as it more closely represents the reality of an individual’s day-to-day life, in which lifestyle behaviors co-occur. Understanding or addressing a single lifestyle behavior may not be enough to fully address mental illnesses with complex, biopsychosocial etiologies. Using rigorous statistical techniques to identify underlying patterns, like latent class analysis, allows for the simultaneous analysis of multiple variables that make up an individual’s lifestyle. Exploring lifestyle as a combination of behaviors also allows us to take a holistic approach to the prevention and treatment of mental illness.

However, grouping behaviors in lifestyle research can be challenging. The term “lifestyle” lacks a standard definition in the research literature. In this review, the specific behaviors comprising a combined lifestyle and their measurement varied considerably across the studies, illustrating a great deal of heterogeneity from study to study. In addition, measurement of lifestyle behaviors was often limited to self-reported single questions instead of validated instruments or objective assessment tools. Given that each study measured multiple behaviors, single items were likely used to reduce participant burden. However, validated instruments, even short-form versions, can reduce measurement bias. For example, regarding diet, only three studies used validated measures of assessment including 24 h recalls [59] or FFQs [48,51].

The substantial heterogeneity observed in the studies limits the ability to generalize the results and draw broad conclusions. For sedentary behavior alone, the studies used various definitions for unhealthy sedentary behavior, including ≥8 h, ≥7.5 h, and ≥90 consecutive minutes, demonstrating the lack of consistency in how lifestyle behaviors are operationalized. Similarly, the studies measured overall diet quality, adherence to dietary patterns such as the Dietary Approaches to Stop Hypertension (DASH) diet, or individual dietary behaviors, such as breakfast consumption and fruit and vegetable intake. These measures capture different aspects of dietary behavior and may not provide comparable representations of overall diet quality. This concern is particularly important when examining combined lifestyle behaviors, as it is unclear whether a single dietary indicator, such as fruit and vegetable consumption, adequately represents “diet” as a component of a broader lifestyle pattern. Assessment of mental health also varied substantially, with 19 different instruments used across the studies to determine various constructs including depression, anxiety, psychological distress, stress, mental well-being, and quality of life. Even when the studies assessed the same mental health construct, various instruments were used, further limiting direct comparisons. As a result, discrepancies in the study findings may reflect variations in the underlying relationships between lifestyle behaviors and mental health, diversity in study populations, or differences in how exposures and outcomes were operationalized. The consistent use of validated assessment tools and development of clear definitions for lifestyle behaviors can help reduce heterogeneity across studies in the research literature and allow for more meaningful comparisons across studies to strengthen the evidence.

Finally, cross-sectional studies cannot measure temporal relationships between lifestyle and mental illness. The relationship between lifestyle and mental illness may also be bidirectional, and therefore these studies may be limited by reverse causation bias. Mental illnesses, like depression, can negatively impact energy levels, motivation, and function. As a result, depression can lead to unhealthy lifestyle behaviors, including poor diet quality, lack of physical activity, sleep disturbances (including both insomnia and excessive sleep), and increased substance use [25,26,27,28,29,30,31,32,33]. Thus, the associations reported in cross-sectional studies cannot determine whether unhealthy lifestyle behaviors contribute to poorer mental health, whether poor mental health contributes to unhealthy lifestyle behaviors, or whether both processes occur simultaneously. This bidirectional relationship is particularly important when interpreting findings from cross-sectional studies, as the combined lifestyle patterns observed may, in part, reflect the impact of existing mental health conditions. Furthermore, the limited number and heterogeneity of randomized controlled trials in emerging adults present a barrier for conducting meta-analyses.

### 4.4. Future Directions

Future research should prioritize longitudinal designs, social and environmental contexts, and the unique needs and challenges of emerging adults. All observational studies included in this review are cross-sectional and therefore cannot address temporal relationships or determine causality. Longitudinal designs can help address this limitation and elucidate the possible bidirectional relationships between lifestyle patterns and mental illness in emerging adults. Existing national datasets, such as Add Health, The National Longitudinal Study of Adolescent to Adult Health, which follows adolescents to adulthood, can be used to explore these relationships [75]. Additionally, future studies can build on these findings by examining whether changes in multiple lifestyle behaviors predict subsequent changes in mental health and whether mental health symptoms similarly predict subsequent changes in lifestyle behaviors.

Future research should also consider the broader social and environmental contexts in which lifestyle behaviors and mental health occur. Social connection is one of the six targeted domains of lifestyle psychiatry but is seldom addressed in lifestyle interventions. Only one observational study included in this review considered the social environment of emerging adults by including whether these individuals experienced negative social situations, such as verbal violence or emotional neglect. Of note, these researchers found that those in the high-risk group who experienced these adverse social experiences had the highest odds of having depression symptoms and suicidal ideation. Furthermore, the limitations of conducting lifestyle interventions in emerging adults, previously presented in this review, include the need for social support for a successful intervention. This support may include the inclusion of group sessions, opportunity to communicate with fellow participants, or education and counseling on how to engage in healthy relationships.

Finally, future intervention research should consider targeting lifestyle behaviors that are tailored to the needs and circumstances of emerging adults. Emerging adults, especially those with mental illness, experience considerable stressors and have competing responsibilities, making it difficult for them to make behavioral changes and engage in research. This population faces unique challenges, particularly misinformation and unrealistic standards perpetuated on social media, financial and employment difficulties in the current economic climate, and a rise in bullying among peers, discrimination, and divisive public discourse. This review highlights diverse lifestyle patterns among emerging adults, reflecting either study heterogeneity or true variation within this population. Future research should investigate interventions that are tailored to distinct lifestyle patterns or individual barriers, as these may be more effective than generalized approaches. In particular, addressing barriers such as limited time, financial constraints, and competing responsibilities may be important for improving intervention engagement and reducing attrition. Furthermore, considering the perspective of emerging adults can help identify these barriers and facilitators to participating in research and adopting a healthy lifestyle. Interventions should not only consider addressing social determinants of health and stress exposures to reduce barriers to engagement and participation but also consider counseling techniques that target personalized behavior change, such as motivational interviewing, to increase intervention effectiveness.

## 5. Conclusions

In this review we identified 17 studies that explored the relationship between combined lifestyle behaviors and mental illness in emerging adults. Our findings suggest that an unhealthy lifestyle may be associated with poorer mental health outcomes in emerging adults. In particular, lifestyles with a large number of unhealthy behaviors, such as high substance use or low physical activity, may be associated with increased burden of mental illness. Although the studies included in this review had considerable heterogeneity, the observational studies reviewed support this conclusion, while the limited numbers of RCTs make it difficult to draw causal conclusions. Given the burden of mental illness in emerging adults, future research is needed to develop interventions that comprehensively target lifestyle behaviors and consider participant burden to ensure effectiveness and feasibility.

## Figures and Tables

**Table 1:** Summary of cross-sectional studies (*n* = 14) and randomized controlled trials (*n* = 3) included in review.

Author/Country	Design	Sample Size	Age (Mean ± Sd, or Range)	Lifestyle Assessment/ Intervention	Mental Health Measures	Clustering Analysis/ Covariates	Results	Limitations
Observational Studies								
Armstrong and colleagues (2024) [48] United States	Cross-sectional	*n* = 222 women	23 ± 5 y	Lifestyle behaviors: • Diet quality via Health Eating Index • Sleep • Tobacco/nicotine use • Alcohol consumption	DASS-21 ^a^	ANOVA, grouping behaviors: • No Unhealthy Lifestyle Behavior • 1 Unhealthy Lifestyle Behavior • 2 Unhealthy Lifestyle Behavior • 3 Or More Unhealthy Lifestyle Behaviors	Those with more concurrent unhealthy lifestyle behaviors had: • ↑ symptoms of depression • ↑ symptoms of anxiety	Cross-sectional, self-reported measures, did not assess impact of COVID-19, not generalizable to other samples, did not consider all lifestyle behaviors (i.e., physical activity)
Armstrong and colleagues (2025) [61] United States	Cross-sectional	*n* = 2019 emerging adults (NHANES ^b^)	22 ± 0.1 y	Lifestyle behaviors: • Diet quality via Dietary Inflammatory Index • Physical activity • Sedentary behavior • Sleep • Tobacco/nicotine use • Alcohol consumption	PHQ-9 ^c^	LCA ^d^ identified 6 male classes and 5 female classes Males: • PID ^e^ but Non-Substance User • Poor Sleep Sedentary Drinkers • Healthy but Drinkers • Healthy • PID Smokers Drinkers • Sedentary Drinkers Females: • PID Smokers Drinkers • Trouble Sleeping • Healthy • PID Poor Sleep • PID Sedentary Covariates considered: age, race, education level, family income, and employment status	Compared to the Healthy group, males in the Poor Sleep Sedentary Drinkers and PID Smokers Drinkers groups had: • ↑ odds of depression Compared to the Healthy group, females in the PID Smokers Drinkers, Trouble Sleeping, and PID Sedentary groups had: • ↑ odds of depression	Cross-sectional, self-reported measures, did not assess impact of COVID-19, did not consider all lifestyle behaviors
Champion and colleagues (2018) [49] Australia	Cross-sectional	*n* = 853 emerging adults	19 ± 0.4 y	Lifestyle behaviors: • Diet (fruit and vegetable intake) • Physical activity • Sedentary behavior • Sleep • Tobacco use • Binge drinking	K-6 ^f^, BSI ^g^	LCA identified 3 classes: • Moderate Risk • Inactive, Non-Smokers • Smokers and Binge Drinkers	Compared to the Moderate-Risk group, Smokers and Binge Drinkers had: • ↑ psychological distress • ↑ symptoms of anxiety and depression Compared to the Moderate-Risk group, Inactive, Non-Smokers had: • ↑ symptoms of depression	Cross-sectional, self-reported measures, high cohort dropout rate, not generalizable to other samples
Collins and colleagues (2023) [50] Australia	Longitudinal cohort	*n* = 458 emerging adults	20–22 y	Lifestyle behaviors: • Diet via DASH ^h^ diet score • Physical activity • Sedentary behavior • Tobacco use • Alcohol consumption • Marijuana and other illicit drug use	DASS-42 ^a^	LCA identified 3 classes: • Healthier Pattern • High Substance Use, Low Physical Activity Pattern • High Substance Use, Low Diet Quality Pattern Covariates considered: baseline mental health, highest level of education, current study status, and income	Compared to the Healthier Pattern, participants in the High Substance Use, Low Physical Activity Pattern had: • ↑ odds of more severe depressive symptoms • ↑ odds of more severe anxiety symptoms • ↑ odds of more severe stress symptoms	Cross-sectional, self-reported measures, measurement inconsistencies at baseline and follow-up, did not assess adherence to patterns overtime
Deasy and colleagues (2014) [51] Ireland	Cross-sectional	*n* = 1112 emerging adults	17–26 y	37-item lifestyle behavior questionnaire: • Diet quality rating • Physical activity • Tobacco use • Alcohol consumption • Marijuana use	GHQ-28 ^i^	Mixed cluster analysis identified 2 groups: • Students With Positive Health Behaviors • Students With Risk Behaviors	Compared to the Students With Positive Health Behaviors, the Students With Risk Behaviors had: • ↑ psychological distress scores (statistical findings were not reported)	Cross-sectional, self-reported measures, not generalizable to other samples
Debbia and colleagues (2019) [52] Spain	Cross-sectional	*n* = 1645 emerging adults	15–24 y	Lifestyle behaviors: • Diet (intake of 16 food items) • Physical activity	Current depression, history of depression, The Goldberg Health Questionnaire-12	Multiple logistic regression, stratified by gender No covariates specified	High psychological/psychiatric vulnerability was associated with: • ↑ number of hours spent sitting in a day • ↓ frequency of fresh fruit consumption • ↓ frequency of breads and cereals consumption • ↑ number of days/week dedicated to intense physical activity	Cross-sectional, self-reported measures, modest variance explained by models
Dong and colleagues (2024) [53] China	Cross-sectional	*n* = 1740 emerging adults	20 ± 1 y	Lifestyle behaviors: • Diet (fruit and vegetable intake) • Physical activity • Sedentary behavior • Smoking status • Alcohol consumption	PHQ-9, GAD-7 ^j^	LCA identified 3 classes for both males and females: • Physical Inactivity, Substance Use, and Insufficient Fruit Intake • Sedentary only • Sedentary, Substance Use, and Unhealthy Diet 1 unique class for males: • Physical Inactivity and Unhealthy Diet 1 unique class for females: • Physical Inactivity and Insufficient Fruit Intake Covariates considered: stratified by sex	Compared to the Sedentary Only group, the Sedentary, Substance Use, And Unhealthy Diet group had: • ↓ anxiety • ↑ depression	Cross-sectional, convenience sample, self-reported measures, not generalizable to other samples, did not consider all lifestyle behaviors
Grant and colleagues (2019) [54] United States	Cross-sectional	*n* = 17,286 emerging adults	18–25 y	Lifestyle behaviors: • Diet (fruit and vegetable intake) • Physical activity • Alcohol consumption	Number of days with poor mental health	LCA identified 5 classes: • Moderates • Unhealthy Eaters • Medium Drinkers • Healthy Eaters/Exercisers • Heavy Drinkers Covariates considered: stratified by sex	For both males and females, compared to Moderates and Healthy Eaters/Exercisers, Medium Drinkers and Heavy Drinkers had: • ↑ number of days with poor mental health	Cross-sectional, self-reported measures, construct of “unhealthy eating” limited to fried potato consumption, type of physical activity was not measured, BMI ^k^ as an indicator of physical health
Jao and colleagues (2019) [55] United States	Cross-sectional	*n* = 105,781 emerging adults	23 ± 6 y	Lifestyle behaviors: • Diet (fruit and vegetable intake) • Physical activity • Cigarette and marijuana use • Binge drinking	Mental health diagnosis, presence of suicidal or self-injurious behaviors, self-reported psychological symptoms	LCA identified 3 classes: • High Alcohol and Drug Use • Low Physical Activity • Low-Risk Behavior	Compared to the other 2 groups, High Alcohol and Drug Use had: • ↑ mental health diagnosis in the past 12 months Compared to the Low-Risk Behavior group, the Low Physical Activity and High Alcohol and Drug Use groups had: • ↑ psychological symptom scores in the past 30 days and past 12 months	Colleges self-participate in NCHA ^l^, cross-sectional, self-reported measures, small effect sizes, binge drinking measure did not include gender-specific definitions by NIAAA ^m^ guidelines
Tran and colleagues (2017) [56] France	Cross-sectional	*n* = 4184 emerging adults	18–20+ y	Lifestyle behaviors: • Diet (rhythm, unbalanced meals, junk food, dieting) • Tobacco and recreational drug use • Alcohol consumption • Binge drinking	Physician screening for depressive or anxiety disorders	Multivariate logistic regression Covariates considered: gender, age	Students who ate junk food and had “bad dietary behavior” had: • ↑ risk for depression	Cross-sectional, self-reported measures, recruitment/sampling bias, not generalizable to other samples
Wickham and colleagues (2020) [57] New Zealand	Cross-sectional	*n* = 1111 emerging adults	18–25 y	Lifestyle behaviors: • Diet (fruit and vegetable intake, fast food, sweets, and soda consumption) • Physical activity • Sleep	CES-D ^n^, Flourishing scale	Hierarchical regression analysis Covariates considered: age (18–25), gender, race/ethnicity, education level, employment status, socioeconomic status, BMI, health condition, supplement use, food exclusion	Sleep quality, sleep quantity, and PA predicted: • ↑ depressive symptoms	Cross-sectional, self-reported measures, did not address shared variance of health behaviors
Xiang and colleagues (2025) [58] China	Cross-sectional	*n* = 6372 emerging adults	College freshman to juniors	Lifestyle behaviors: • Diet (breakfast, fast food, sweetened beverages, and milk, soymilk and yogurt) • Physical activity • Sedentary behavior • Sleep • Smoking status • Alcohol consumption • Sex behaviors • Adverse experiences	CES-D, Suicidal ideation	LCA identified 4 classes • Low-risk class • Moderate-risk class • High risk (substance abuse/unprotected sex) • High risk (adverse experience [verbal abuse and emotional neglect]) Covariates considered: gender, grade, family structure, sickness absence, socioeconomic level	Compared to the low-risk class, all other groups had: • ↑ odds of having depressive symptoms • ↑ odds of having suicidal ideation	Cross-sectional, self-reported measures
Xie and colleagues (2023) [59] United States	Cross-sectional	*n* = 2268 emerging adults (NHANES)	18–24 y	Lifestyle behaviors: • Diet quality via Health Eating Index • Physical activity • Sedentary behavior • Sleep • Cigarette use • Alcohol consumption	PHQ-9	LCA identified 4 classes • Unhealthy But Non-Substance Use • Healthy But Sleepless And Drinking • Unhealthy Lifestyle • Healthy But Sedentary Covariates considered: household income, education level, insurance status, NHANES cycle	Compared to the Unhealthy Lifestyle group, all other groups had: • ↓ odds of having depressive symptoms • ↓ depression severity	Cross-sectional, self-reported measures, did not assess impact of COVID-19, did not distinguish employed students vs. unemployed non-students, possibly biased measure of cigarette use.
Ye and colleagues (2015) [60] China	Cross-sectional	*n* = 2422 emerging adults	20 ± 1 y	Lifestyle behaviors: • Diet (fruit and vegetable intake) • Physical activity • Sleep • Smoking status • Alcohol consumption • Internet addiction disorder	SDS ^o^, SAS ^p^	Cluster analysis identified 2 clusters: • Cluster 1 (healthier behaviors) • Cluster 2 (unhealthier behaviors) Covariates considered: university, age, sex, BMI and maternal education	Compared to Cluster 1, participants in Cluster 2 had: • ↑ odds of depression • ↑ odds of anxiety	Cross-sectional, self-reported measures, not generalizable to other samples
Randomized Controlled Trials								
Ashton and colleagues (2017) [62] Australia	Randomized controlled trial	*n* = 50 men	18–25 y	Weekly 1 h in-person sessions, 3 months: • 40 min: Physical activity • 10 min: Healthy eating education • 10 min: Stress relief and well-being Control: • Usual lifestyle	K-10 ^f^, DASS-21, MHC-SF ^q^, Q-LES-Q ^r^	Covariates considered: • BMI, physical activity, and proportion of energy from energy-dense, nutrient-poor foods	Compared to control • No significance compared from baseline to 3 months in psychological distress, mental health, mental illness, or quality of life	Small sample size, short duration, self-reported, over-representation of higher education, full-time students, follow-up measures conducted during examination period
Duan and colleagues (2017) [63] China	Randomized controlled trial	*n* = 142 emerging adults	17–24 y	1 20 min web-based session per week, 8 weeks: • Week 1–4: Physical activity • Week 5–8: Fruit and vegetable intake Control: • Usual lifestyle	Hong Kong WHOQOL-BREF ^s^ questionnaire, Chinese CES-D scale	Covariates considered: • Baseline physical activity and fruit and vegetable intake	Compared to control: • No significant differences between groups for depression • ↑ quality of life	High dropout rate, low engagement, short duration, low baseline depression, self-reported
Kaseva and colleagues (2015) [64] Finland	Secondary data analysis of a randomized controlled trial	*n* = 457 emerging adults	20 y	2 sessions/year from infancy to age 20: • Nutritionist-delivered diet counseling • Smoking prevention, physical activity, limiting alcohol and drug use by age 8 Control: • Standard healthcare education	BDI-II ^t^, and a psychological well-being survey	Covariates considered: • Sex, socioeconomic status	Compared to the control: • No significant differences between groups for depression or psychological well-being	Loss to follow-up, did not measure change in psychological well-being over time

^a^ Depression Anxiety Stress Scale-21, 42; ^b^ National Health and Nutrition Examination Survey; ^c^ Patient Health Questionnaire-9; ^d^ Latent Class Analysis; ^e^ Pro-inflammatory diet; ^f^ Kessler-6, 10; ^g^ Brief Symptoms Inventory; ^h^ Dietary Approaches to Stop Hypertension; ^i^ The General Health Questionnaire-28; ^j^ General Anxiety Disorder-7; ^k^ Body Mass Index; ^l^ National College Health Assessment; ^m^ National Institute on Alcohol Abuse and Alcoholism; ^n^ Center for Epidemiological Depression Scale; ^o^ Self-rating Depression Scale; ^p^ Self-rating Anxiety Scale; ^q^ Mental Health Continuum-Short form; ^r^ Quality of Life, Enjoyment, and Satisfaction Questionnaire; ^s^ World Health Organization Quality of Life-BREF; ^t^ Beck Depression Inventory-II.

## Data Availability

No new data were created or analyzed in this study. Data sharing is not applicable to this article.

## Supplementary Materials

The following supporting information can be downloaded at: https://www.mdpi.com/article/10.3390/nu18172797/s1. Figure S1: Flow diagram of study screening and selection; Table S1: Measurement and analysis of lifestyle behaviors in observational studies [48,49,50,51,52,53,55,56,58,59,60,61].
