# Supplementary material for: The Relationship Between Combined Lifestyle Behaviors and Mental Illness in Emerging Adults: A Narrative Review
Source: Nutrients. 2026 Aug 27;18(17):2797. doi: 10.3390/nu18172797 (PMC13567683; doi:10.3390/nu18172797)
Supplement: Supplementary file 1 [file nutrients-18-02797-s001.zip › nutrients-4483489-supplementary.pdf]

Supplementary Materials

Figure S1. Flow Diagram of Study Screening and Selection

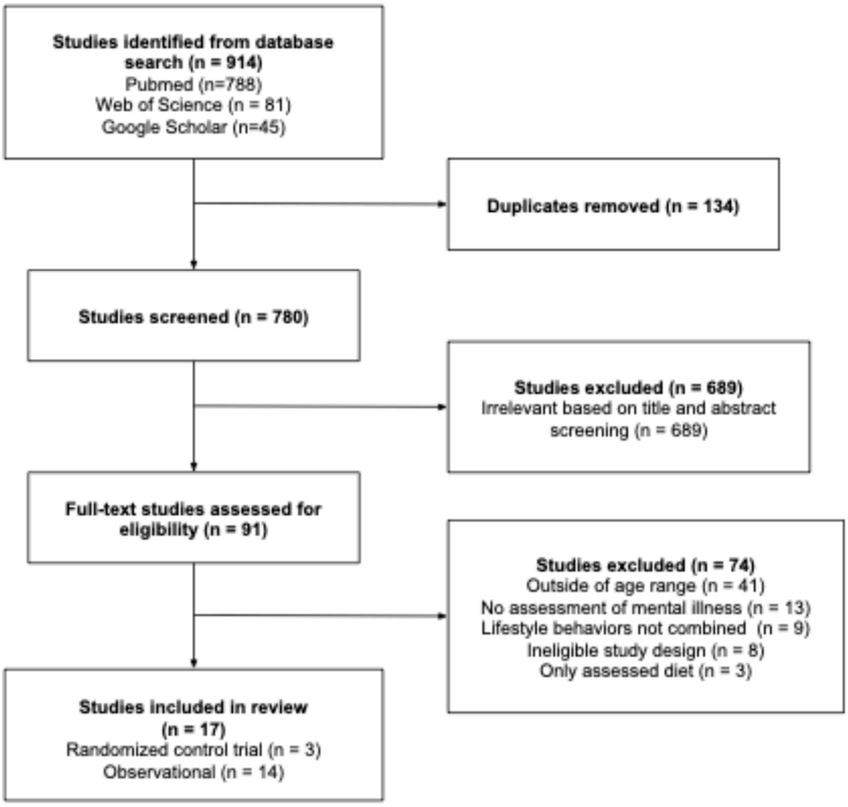

Table S1. Measurement and Analysis of Lifestyle Behaviors in Observational Studies

**Author/ Country**	**Diet**	**Physical Activity and Sedentary Behavior**	**Sleep**	**Alcohol Consumption**	**Smoking Status**	**Other**
Armstrong and colleagues (2024) [48] United States	Healthy Eating Index:<br>• Good quality diet: Healthy Eating Index ≥80<br>• Moderate quality diet: Healthy Eating Index 51-79<br>• Moderate quality diet: Healthy Eating Index ≤50		US[a] Sleep Foundation Guidelines<br>• Inadequate sleep: <7 hours<br>• Adequate sleep: 7-9 hours	Dietary Guidelines for Americans:<br>• Rec[b]: ≤1 drink/day for women or ≤2 drinks/day for men<br>• Excessive intake: >1 drink/day for women or >2 drinks/day for men	• Current smoker vs. not current smoker	
Armstrong and colleagues (2025) [61] United States	Dietary Inflammatory Index:<br>• Pro-inflammatory diet: Dietary Inflammatory Index ≥0<br>• Anti-inflammatory diet: Dietary Inflammatory Index <0	US Physical Activity Guidelines:<br>• Rec: ≥150 minutes of moderate activity or 75 minutes of vigorous activity each week<br><br>WHO[c] Sedentary Behavior Guidelines:<br>• Sedentary: >7.5 hours/day<br>• Non-Sedentary: ≤7.5 hours/day	Sleep Duration: US Sleep Foundation Guidelines<br>• Poor sleep: <7 hours<br>• Adequate sleep: 7-9 hours<br><br>Sleep Quality:<br>• Trouble sleeping: ever told a doctor you had trouble sleeping	Dietary Guidelines for Americans:<br>• Nondrinkers: ≤1 drink/day for women or ≤2 drinks/day for men<br>• Drinkers: >1 drink/day for women or >2 drinks/day for men	Smoking history<br>• Smokers: smoked at least 100 cigarettes in a lifetime<br>• Nonsmokers: has not smoked at least 100 cigarettes in a lifetime	
Champion and colleagues (2018) [49] Australia	Australian Dietary Guidelines:<br>• Poor fruit and vegetable intake: <2 servings/day<br>• Sufficient fruit and vegetable intake: 4-5 servings/day	AUS[d] Physical Activity Guidelines<br>• Rec: ≥150 minutes of moderate activity or 75 minutes of vigorous activity each week<br><br>Sedentary Behavior:<br>• Sedentary: >8 hours/day<br>• Non-Sedentary: ≤8 hours/day	AUS Sleep Foundation Guidelines<br>• Inadequate sleep: <6 hours<br>• Adequate sleep: 6-11 hours	AUS National Health and Medical Research Council Guidelines<br>• Binge drinking: ≥5 drinks on one occasion	• Using tobacco >1 time vs. ≤1 using tobacco	
Collins and colleagues (2023) [50]	DASH[e] Diet Score:<br>• Score ranges 0-11	AUS Physical Activity Guidelines and WHO		AUS National Health and Medical Research Council Guidelines:	• Current smoker vs. not current smoker	• Frequency of marijuana and other illicit drug use

Australia	• Scores categorized into quartiles	Physical Activity Guidelines:<br>• Rec: ≥150 minutes of moderate activity or 75 minutes of vigorous activity each week		• Low intake >0 and <2 drinks<br>• Moderate intake ≥2 and <4 drinks<br>• High intake ≥4 drinks		
Deasy and colleagues (2014) [51] Ireland	Lifestyle Behaviour Questionnaire:<br>• Item "Please rate your diet"<br>• Healthy diet: "strongly agree" and "agree"<br>• Unhealthy diet: "neutral", "disagree" and "strongly disagree"	Physically active individuals:<br>• Physical activity >4 days/week		• ≤2 days drinking/week vs. >3 days drinking/week	• Current smoker vs. not current smoker	• Marijuana use in the last 6 months
Debbia and colleagues (2019) [52] Spain	• Frequency of consumption of fresh fruit (juices excluded), meat, eggs, fish, pasta, rice and potatoes, bread and cereals, vegetables, legumes, cured meats, dairy products, sweets, soft drinks (with sugar), fast food, appetizers and snacks, fresh fruit or vegetable juices<br>• Low<br>• Medium<br>• High	Cut points not specified				
Dong and colleagues (2024) [53] China	Dietary Guidelines for the Asia Pacific Region:<br>• 5 or more days: high fruit and vegetable intake<br>• <5 days: low fruit and vegetable intake	WHO Physical Activity Guidelines:<br>• Rec: ≥150 minutes of moderate activity or 75 minutes of vigorous activity each week<br><br>Sedentary Behavior:		• 0 drinks/month vs. ≥1 drink/month	• Current smoker vs. not current smoker	

	• Daily breakfast: regular<br>• ≤ daily breakfast: irregular	• ≥90 minutes consecutively/day				
Jao and colleagues (2019) [55] United States	US Dietary Guidelines:<br>• Insufficient fruit and vegetable intake: <5 servings	US Physical Activity Guidelines:<br>• Rec: ≥150 minutes of moderate activity or 75 minutes of vigorous activity each week		• Binge drinking: ≥5 drinks in one sitting	• Current smoker vs. not current smoker	• Frequency of marijuana use in the last 30 days
Tran and colleagues (2017) [56] France	Poor dietary behavior in the past 30 days including:<br>• Irregular rhythm of meals<br>• Unbalanced meals<br>• Eating junk food<br>• On a diet<br>• Irregular rhythm of meals or unbalanced meals			• <4 days drinking in the last 30 days vs. ≥4 days drinking in the last 30 days<br><br>• Binge drinking: ≥5 drinks in one sitting	• Nonsmokers: 0 cigarettes per day<br>• Occasional to regular smokers: 1-10 cigarettes per day<br>• Frequent smokers: ≥11 cigarettes per day	
Xiang and colleagues (2025) [58] China	Poor dietary behavior:<br>• Skipping breakfast at least once within the last week<br>• Sweetened beverages >1x/day<br>• Fast food >1x/day<br>• Milk, soymilk and yogurt <1x in the previous week	Physical inactivity:<br>• <2 days of at least 60 min per day of moderate or vigorous intensity physical exercises in the last week<br><br>Sedentary Behavior:<br>• >2 hours sitting, watching TV, playing computer games or using social media on an average day in the last week	• Insufficient sleep: <7 hours/day	• Drinking at least 1 beverage once in the last 30 days	• Smoking at least once in the last 30 days	Adverse experiences:<br>• Verbal violence and emotional neglect in the last 30 days<br><br>Sex behaviors:<br>• Sex without a condom in the last 30 days
Xie and colleagues (2023) [59] United States	Healthy Eating Index:<br>• Score range 0-100<br>• Scores in the top two quintiles considered healthy	WHO Physical Activity Guidelines:<br>• Rec: ≥150 minutes of moderate activity or 75 minutes of vigorous activity each week	• Healthy: 6-8 hours	Dietary Guidelines for Americans:<br>• Rec: ≤1 drink/day for women or ≤2 drinks/day for men	• Smoking at least 100 cigarettes in a lifetime	

		Sedentary Behavior:<br>• Sedentary: >7.5 hours/day<br>• Non-Sedentary: ≤7.5 hours/day		• Excessive intake: >1 drink/day for women or >2 drinks/day for men		
Ye and colleagues (2015) [60] China	Poor dietary behavior:<br>• Eating fruits or vegetables ≤3x per week during the last 30 days	Physical activity:<br>• Engaging in sport or vigorous activity ≥3 days for 30 minutes	Pittsburgh Sleep Quality Index:<br>• Score >5 indicates sleep disturbance	• ≤2 times drinking/week vs. >3 times drinking/week	• Current smoker vs. not current smoker	Young's 20-item Internet Addiction Test:<br>• Internet addiction disorder: score ≥50

[a] United States
[b] Recommendation
[c] World Health Organization
[d] Australian
[e] Dietary Approaches to Stop Hypertension
[f] Body Mass Index
